# Robotic hernia surgery I. English version: Robotic inguinal hernia repair (r‑TAPP). Video report and results of a series of 302 hernia operations

**DOI:** 10.1007/s00104-021-01446-1

**Published:** 2021-06-29

**Authors:** Michaela Ramser, Johannes Baur, Nicola Keller, Jan F. Kukleta, Jörg Dörfer, Armin Wiegering, Lukas Eisner, Ulrich A. Dietz

**Affiliations:** 1 Department of Visceral, Vascular and Thoracic Surgery, Cantonal Hospital Olten (soH), Baslerstrasse 150, 4600 Olten, Switzerland; 2 Department of General, Visceral and Vascular Surgery, Cantonal Hospital Baden, Im Engel 1, 5404 Baden, Switzerland; 3 Hernienzentrum Zurich, Grossmuensterplatz 9, 8001 Zurich, Switzerland; 4 Department of General, Visceral, Transplant, Vascular and Pediatric Surgery, University Hospital Wuerzburg, Oberduerrbacher Straße 6, 97080 Wuerzburg, Germany

**Keywords:** Groin hernia, Endoscopic groin hernia repair, Learning curve, Transverse fascia, Seroma, Leistenhernie, Minimalinvasive Leistenhernienversorgung, Lernkurve, Fascia transversalis, Serom

## Abstract

The treatment of inguinal hernias with open and minimally invasive procedures has reached a high standard in terms of outcome over the past 30 years. However, there is still need for further improvement, mainly in terms of reduction of postoperative seroma, chronic pain, and recurrence. This video article presents the endoscopic anatomy of the groin with regard to robotic transabdominal preperitoneal patch plasty (r‑TAPP) and illustrates the surgical steps of r‑TAPP with respective video sequences. The results of a cohort study of 302 consecutive hernias operated by r‑TAPP are presented and discussed in light of the added value of the robotic technique, including advantages for surgical training. r‑TAPP is the natural evolution of conventional TAPP and has the potential to become a new standard as equipment availability increases and material costs decrease. Future studies will also have to refine the multifaceted added value of r‑TAPP with new parameters.

**Video online:**

The online version of this article contains one video. The article and the video are online available (10.1007/s00104-021-01446-1). The video can be found in the article back matter as “Electronic Supplementary Material”.

## Abstract (de)

Die Versorgung von Leistenhernien mit offenen und minimal-invasiven Verfahren hat in den vergangenen 30 Jahren einen vom Ergebnis her gesehen hohen Standard erreicht. Allerdings besteht noch Bedarf an einer weiteren Reduktion der postoperativen Serome, chronischen Schmerzen und des Rezidivs. In diesem Videobeitrag wird die endoskopische Anatomie der Leiste im Hinblick auf die robotische transabdominelle präperitoneale Patchplastik (r‑TAPP) dargestellt und die Operationsschritte der r‑TAPP als Video illustriert. Es werden die Ergebnisse einer Kohortenstudie an 302 konsekutiven Hernien, die mittels r‑TAPP operiert wurden, vorgestellt und hinsichtlich des Mehrwerts der robotischen Technik – auch in der Weiterbildung – diskutiert. Die r‑TAPP ist die natürliche Weiterentwicklung der konventionellen TAPP und hat das Potenzial, bei zunehmender Geräteverfügbarkeit und Reduktion der Materialkosten zu einem neuen Standard zu werden. Künftige Studien werden den vielseitigen Mehrwert der r‑TAPP auch mit neuen Parametern verfeinern müssen.

## Background

Laparoscopic inguinal hernia repair using TAPP (transabdominal preperitoneal patch plasty) began 30 years ago (1991). In its early days, TAPP had to compete against very good open procedures, the operation was complex and expensive laparoscopic systems had to be acquired. The opposing arguments were strong: in the Shouldice Clinic (Canada) inguinal hernias had been operated since the 1950s with excellent results, without mesh and better than after Bassini; in the Lichtenstein Institute (USA) the tension-free mesh repair had been perfected in 1985. Shouldice and Lichtenstein were performed under local anesthesia, were inexpensive and very convincing (see also the video article in *Der Chirurg* by Dietz et al. 2016; [1]).

How could laparoscopic TAPP establish itself against such strong arguments? As it is so often the case with new developments in surgery, we owe TAPP not to robust preclinical data but to visionary pioneers who recognized the potential benefits of minimally invasive endoscopic work [2]. It was not until years later that the results of the “optimal TAPP” (working group of Prof. Reinhard Bittner, Stuttgart) versus the “perfect Lichtenstein” (working group of Prof. Henrik Kehlet, Copenhagen) were published: with a comparable recurrence rate but significantly less chronic pain after TAPP than after Lichtenstein (*p* = 0.018; odds ratio [OR] 0.45; confidence interval [CI] 0.23–0.87; [3]).

However, recurrence after TAPP is a problem that requires further improvement; an exemplary study showed a recurrence rate of about 3.5% for endoscopic repairs [4]. The HerniaSurge guideline (2018) draws attention to the fact that poor quality of hernia repair is one of the preventable risk factors for recurrence, which is why continuing education, standardization, and attention to the learning curve are so important [5]. Suboptimal results can be of great socioeconomic relevance—measured by the frequency of these operations—not least because quality of life and body enhancement have gained an important status in the population. This leads to two conclusions

- In the never-ending cycle of validation and falsification, after 30 years of TAPP, new technologies must be given space to further improve outcomes, and
- It is in the nature of progress that even small improvements in outcomes today require greater effort than improvements in outcomes in the past.

Robotic TAPP (r-TAPP) is thus the natural evolution of conventional TAPP. Empowered by the stability and magnification of the image, the ergonomic working in a wide intra-abdominal space and the intuitive working possibilities of the precision instruments, surgeons regain a more natural handling regarding tissue preparation and greater freedom in suturing and tying than was previously known in minimally invasive procedures.

In this paper, the steps of r‑TAPP are presented and illustrated with results from our own patient collective. The posterior anatomy of the groin region is reviewed and discussed in the context of robotic surgery in the accompanying video report.

## Indications and contraindications

The indications for r‑TAPP are basically similar to those for conventional TAPP [5]. The endoscopic advantages of inspecting the bowel in cases of incarcerated hernias are also valid for robotics. What is new with robotics is that morbid obesity and age have less influence on the choice of procedure than with conventional TAPP. Robotics has some noticeable advantages that go far beyond ergonomics, degrees of freedom of the instruments, image stability and immersion view, for example:

- Since the ports are fixed to the robotic arms, these act against the weight of the abdominal wall, as in the earlier lift laparoscopy, and allow a markedly improved overview in obese patients.
- If necessary, lower intraperitoneal pressure, e.g., 6–8 mm Hg, can be used for patients with cardiopulmonary impairment.
- The distance between the ports and the target organ is always constant (not dependent on the level of the belly button); thus, the working conditions are constantly reproducible even with different body biotypes.

This also facilitates interventions after previous abdominal surgeries, in the presence of stomas, in recurrent hernias or after prostate resections.

The indication for surgery is challenging in the case of preoperative pain history or an increased pain risk profile; often, preoperative analgesic treatment should be considered, as preoperative pain correlates with chronic pain [3]. This is particularly important in young patients and athletes; in some cases, magnetic resonance imaging (MRI) exclusion of other causes of pain (e.g., adductor tendinitis, symphysitis, lumbar spine syndrome) should be considered.

## Informed consent

Basically, the same approach to informed consent applies as for conventional TAPP. The minimally invasive procedure is presented, with the covering of all potential hernia orifices with mesh as well as the option to treat the groin region of the opposite side or concomitant Spieghel hernias. Postoperative complications such as postlaparoscopic shoulder pain, urinary retention, postoperative bleeding, seroma formation and the occurrence of chronic pain are discussed with the patient [6]. The puncture site of the Veres needle on the left subcostal area (the needle was invented by the Hungarian internist János Veres, 1903–1979) and the shaving of the abdomen and the right thigh (for the neutral electrode) are disclosed. The available results of recurrence rates of conventional endoscopic repair are mentioned (about 3.5% over 5 years). The implantation of an MRI-visible nonabsorbable large-pored mesh is explained to the patient. We also discuss the use of the DaVinci Xi with the patients and explain that it is not an actual robot, but a precision instrument that is guided exclusively by the surgeons.

Patients with risk profile for chronic pain (e.g. known chronic pain syndrome, young women or Mediterranean ethnicity) are given a prescription for pregabalin, starting on the eve of surgery and continuing for another 3 days, together with the usual pain medication (paracetamol and ibuprofen). Patients are advised about options for postoperative scar management.

## Anesthesia and patient positioning

Before the operation, in the day clinic, there is a final talk with the patient, the informed consent sheet is checked and the side to be operated on is marked on the awake patient’s skin with a waterproof pen. The patient is placed in the supine position on an antislip mat (Pink Pad, Xodus Medical, New Kensington, PA, USA) on the operating table (Trumpf Medical, Saalfeld, Germany), one arm is moved out for anesthesia (e.g., the ipsilateral patient arm for positioning the robot), the face and ventilation tube are protected with a metal frame mounted on the operating table. When using the DaVinci Xi system (Intuitive Surgical, Sunnyvale, CA, USA), the patient side on which the robot is positioned is not relevant. In the vast majority of cases, a head-down position of 10° Trendelenburg is sufficient; in very obese patients or inguinoscrotal hernias, 15° Trendelenburg is helpful. The procedure is performed under general anesthesia; relaxation must be optimal until the end of the procedure and until undocking of the robotic system; if necessary, the neuromuscular blockade is antagonized at the end of the procedure. Extubation is not performed in the operating room so that it can be cleaned and re-equipped for the next planned procedure without loss of time.

## Overview of endoscopic groin anatomy

**Fig. 1:**
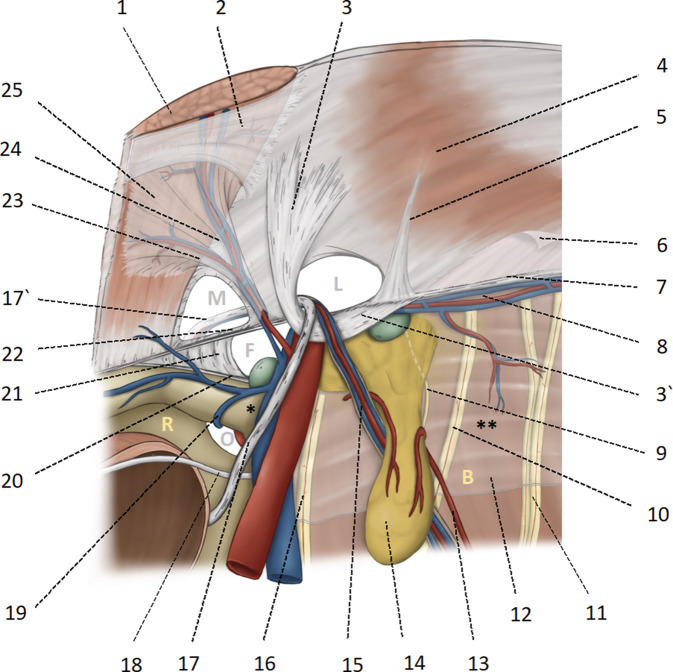
Anatomical basis of the myopectineal orifice (Fruchaud) or inner groin region. *1* Rectus abdominis; *2* Posterior rectus sheath with the arcuate line; *3* and *3’* Hesselbach’s ligament; *4* transverse muscle; *5* Henle’s ligament; *6* intermediate and endoabdominal fascia, respectively; *7* iliopubic tract; *8* A. and V. circumflexa iliaca interna; *9* genital branch of the genitofemoral nerve; *10* femoral branch genitofemoral nerve; *11* lateral femoral cutaneous nerve; *12* iliac fascia; *13* vascular supply of the lipoma from proximal and distal; *14* lipoma; *15* testicular vessels; *16* femoral nerve; *17* and *17’* deferent duct (in women the round ligament of uterus); *18* obliterated umbilical artery; *19* obturator vein; *20* pectineal ligament of Cooper; *21* lacunar ligament of Gimbernat; *22* the inner part of the inguinal ligament corresponds approximately to the iliopubic tract; *23* medial branch of the epigastric vessels, also known as corona mortis according to Hesselbach and Cooper; anastomoses between the retropubic vessels and the corona mortis are known as Bendavid’s circulus venosus; *24* inferior epigastric vessels; *25* fascia of the rectus abdominis. *M* medial hernia in Hesselbach’s triangle, *L* lateral hernia, internal inguinal ring, *F* femoral hernia, *O* foramen obturatorium, * triangle of doom (caveat: vascular injury), ** triangle of pain (caveat: nerve injury), *R* space of Retzius, *B* space of Bogros. Femoral and iliac lymph nodes are shown in *green*

According to the French surgeon Henri Fruchaud (1894–1960), the posterior (in our times endoscopic) analysis of the groin provides a “panoramic view” of the *myopectineal orifice*, which is inseparable as an anatomical entity and includes the three potential hernial orifices medial, lateral, and femoral (Fig. 1). In each r‑TAPP, all three potential hernial orifices are systematically visualized and explored [7].

The *peritoneum* of the inguinal region adheres to the transversus abdominis muscle on the lateral side and to the posterior rectus sheath on the medial side with loose strands of connective tissue and partly through a layer of fatty tissue. The exact course of the endoabdominal fascia or the intermediate fascia as well as the fascia of the rectus muscle below the arcuate line must first be redefined in anatomical studies; there is still no conclusive clarity here, neither on the part of anatomy nor embryology. The leading structure of the endoscopic groin anatomy is the *inferior epigastric artery* (Fig. 1/24). Medial to the inferior epigastric artery, lateral to the rectus abdominis muscle and cranial to the inguinal ligament is the *Hesselbach triangle* (named after Franz Caspar Hesselbach, 1759–1816); it is lined by the transversalis fascia and is the site of medial (direct) hernias (Fig. 1/M). It is likely that what surgeons call “transversalis fascia” is the medial insertion aponeurosis of the transversus abdominis muscle, while the actual “transversalis fascia” is most likely part of the larger endoabdominal fascia [8]. In the current text, the term transversalis fascia refers to the historical meaning of the posterior wall of the inguinal canal in Hesselbach’s triangle, as is common among surgeons. The bulge of the transversalis fascia in medial hernias forms the so-called “*outer sac*”. In this area there are often venous anastomoses between the inferior epigastric vein and the retropubic veins (rectusial veins), the so-called “venous circulus” of Robert Bendavid (1940–2019); the vascular branches running medially from the epigastric vessels above the triangle of Hesselbach are the actual *corona mortis* (Fig. 1/23). In emergency herniotomy in the centuries before modern hernia repair, the hernial ring incision was made “from the bottom up” to widen the inguinal ring and free the incarcerated intestine, whereby in medial hernias the internal bleeding at this point often led to death and the vessels running there therefore became known as the corona mortis. The Würzburg prosector Hesselbach (1759–1816) invented a surgical clamp with a screw cap to stop bleeding at the corona mortis. Even if the retropubic or rectusial veins appear dangerous to endoscopic surgeons, they do not correspond to the original corona mortis.

Lateral to the inferior epigastric artery is the *internal inguinal ring* (Fig. 1/L), confined inferiorly by the inguinal ligament or iliopubic tract and superiorly by the transversus and obliquus internus muscles. Lateral (indirect) hernias emerge here. It is not uncommon to find caudal Spieghel hernias (named after the Brussels anatomist Adrian van der Spieghel, 1578–1625) slightly lateral to the latter. In lateral hernias, the peritoneal hernia sac, together with the *testicular vessels* (Fig. 1/15) and the *deferent duct* (Fig. 1/17), runs through the inguinal canal; in women, the *round ligament of uterus* runs through the inguinal canal. The *genital branch of the genitofemoral nerve* (Fig. 1/9) passes through the inguinal canal in only about 14% of cases; it usually perforates the iliopubic tract (Fig. 1/22) or passes cranially of it through the abdominal wall and finds its way to the cremasteric fibers [9]. In the inguinal canal along the hernia sac, the genital branch of the genitofemoral nerve runs in the “blue line” known from open hernia repair, adjacent to a testicular vein [10]. The peritoneum of the lateral inguinal hernia forms the so-called “*inner sac*”. In 20–70% of patients there is a *cord lipoma* (Fig. 1/14), which is more correctly a preperitoneal fat prolapse and not an actual lipoma; the lipoma of preperitoneal origin is cranially pedunculated (Fig. 1/13) and usually projects into the inguinal canal laterally of the hernial sac and the spermatic cord; it receives its blood supply from proximal to the inguinal ring [11]. In approximately 8% of patients, there is only a lipoma, without a peritoneal hernia sac; this finding is classified as a European Hernia Society (EHS) L1 hernia [12]. There are also spermatic cord lipomas that fill the inguinal canal “like a string of pearls” and without a recognizable vascular pattern; these probably arise from the fatty tissue of the spermatic cord.

The *external iliac artery and vein* run with the *femoral nerve* (Fig. 1/16) and eventually with *lymph nodes* (Fig. 1/green) through the femoral canal (Fig. 1/F) under the iliopubic tract into the thigh. Medially, the femoral canal is demarcated by the *lacunar ligament* (named after the Spanish surgeon Don Antonio de Gimbernat y Arbós, 1734–1816; Fig. 1/21), here the femoral hernia is formed (Fig. 1/F). The lacunar ligament connects the inguinal ligament to the *pectineal ligament of Cooper* (named after the London surgeon Sir Astley Paston Cooper, 1768–1841). Below the inguinal ligament or iliopubic tract and the inner inguinal ring are iliac lymph nodes (Fig. 1/green). The space between the symphysis and the urinary bladder is known as the *space of Retzius* (named after the Swedish anatomist Anders Adolf Retzius, 1796–1860; Fig. 1/R); recent anatomical studies define the preperitoneal space of Retzius as the space between the urogenital fascia (which covers the urinary bladder) and the transversalis fascia (which is part of the endoabdominal fascia and should not be confused with the fascia of the transverse muscle; [8]). Below the pubic bone runs the *obturator canal* (Fig. 1/O). The space of Retzius opens laterally of the iliac vessels into the preperitoneal *space of Bogros* (named after the French anatomist Annet-Jean Bogros, 1786–1823; Fig. 1/B; [13]); the *genitofemoral nerve* (Fig. 1/10; with its genital and femoral branches) and the *lateral femoral cutaneous nerve* (Fig. 1/11), are generally to be found under the iliac fascia (Fig. 1/12), which protects them; the *iliac fascia* and the above-mentioned nerves must remain undamaged during dissection [9, 14, 15].

During endoscopic preparation as part of r‑TAPP, the ilioinguinal and iliohypogastric nerves are not visible; they run out of the pelvis cranial to the anterior superior iliac spine and enter the space between the oblique internal and external muscles in the groin region.

## Surgical steps

Starting with the WHO team time-out, the planned steps are repeated and possible deviations are discussed using the standardized intraoperative checklist (Fig. 2). The pneumoperitoneum is established using a Veres needle and the three working ports are positioned in a standardized way (Fig. 3; additional material online, video sequence 00:58–02:04 min). Round view by means of a diagnostic laparoscopy. Docking of the robotic system and table positioning in 10° head-down position (Trendelenburg). The DaVinci Xi (Intuitive Surgical, CA, USA) and the operating table (Trumpf Medical, Saalfeld, Germany) are coupled via Bluetooth; if necessary, the table can be repositioned (e.g., in very obese patients) with the robot still docked during the operation. After docking to the ports, the robot arms hovering above the patient are slightly displaced towards the ceiling (via the port clutch button), which expands the radius of the abdomen and allows working with lower pneumoperitoneum pressure at the same intra-abdominal volume (8–12 mm Hg). Since the robotic memory pays attention to the so-called remote center of the ports (black ring on the port shaft), the abdominal wall is not damaged by the movements of the instruments. We work with the DaVinci Xi on two surgical consoles. The monopolar scissors (Hot Shears MCS), grasping forceps (Prograsp Forceps) and the needle holder with integrated scissors (Mega SutureCut Needle Driver), as well as a 30° optic are used as standard instruments. As an alternative to the Prograsp Forceps, a bipolar grasping forceps (Fenestrated Bipolar Forceps or Maryland Bipolar Forceps) can be used.

**Fig. 2:**
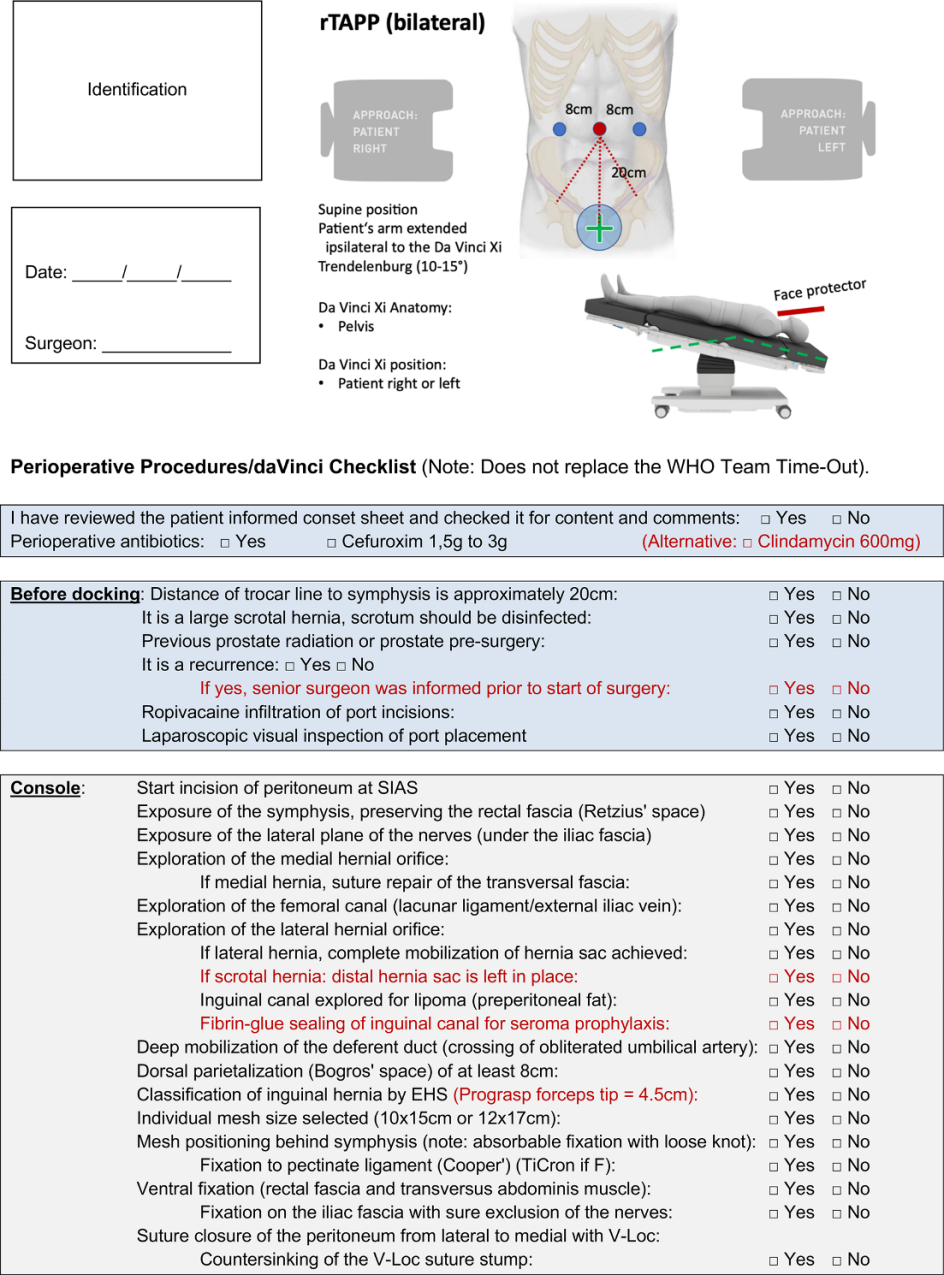
Intraoperative checklist. *SIAS* spina iliaca anterior superior (anterior superior iliac spine), *EHS* European Hernia Society, *OR* operating room, *rTAPP* robotic inguinal hernia repair, *CIRS* critical incident report system, *AVOS* Ambulant-vor-Stationär (outpatient surgery), *S‑DRG* Swiss diagnosis-related group

**Fig. 2:**
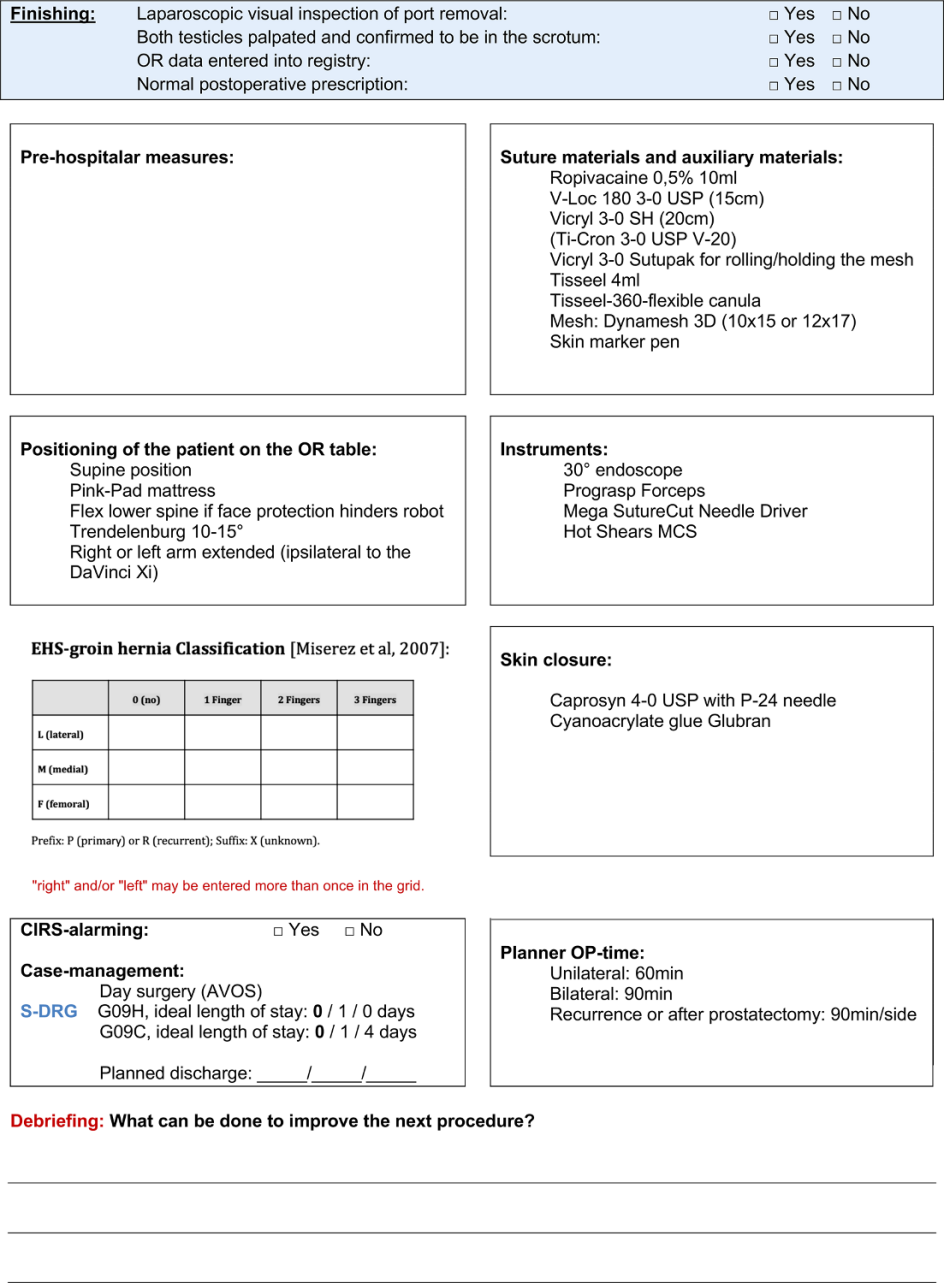
(Continued)

**Fig. 3:**
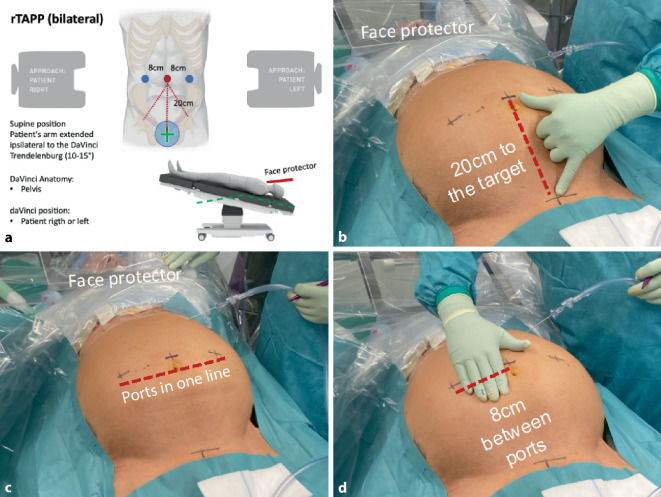
Positioning of ports for robotic inguinal hernia repair (r-TAPP). **a** Excerpt from Kantonsppital Olten’s r‑TAPP surgical checklist with summary of patient’s positioning on the operating room table (very important: pay attention to face protection), approach of the DaVinci Xi to the patient and illustration of target alignment (*green cross* *in* *blue circle*); **b** working distance between the ports and the target organ: 20 cm; **c** the ports are positioned in one line; **d** distance between the ports: 7–8 cm (depending on patient size)

**Fig. 4:**
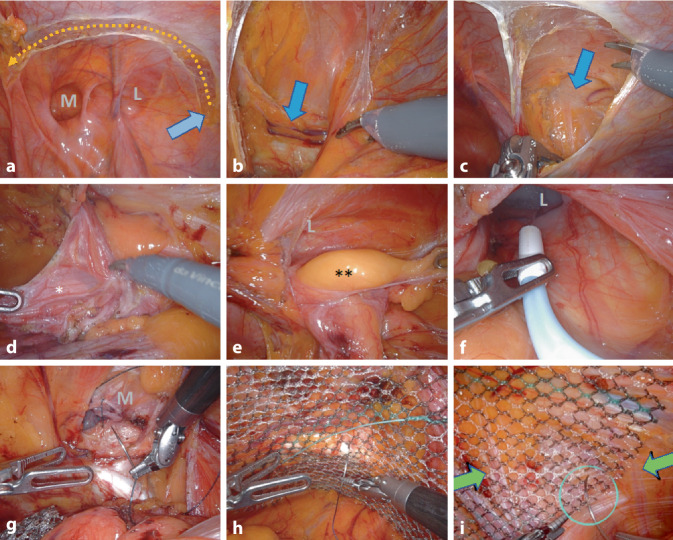
Surgical steps of robotic inguinal hernia repair (r-TAPP). **a** Opening of the peritoneum, starting laterally in projection of the anterior superior iliac spine (*blue arrow*), in a wing-like arc medially to the lateral umbilical fold. **b** Visualization of the pubic bone (*blue arrow*). **c** Visualization of the plane of the nerves under the iliac fascia, laterally. **d** Preparation of the hernia, in this case a lateral hernia, with monopolar dissection and separation of the hernia sac (*) from the deferent duct and the testicular vessels. **e** Exploration of the inguinal canal for the presence of lipoma (**) or preperitoneal fatty tissue. **f** In the case of long hernia sacs, the inguinal canal is sprayed with fibrin glue for seroma prophylaxis. **g** In the case of medial hernias, the transversalis fascia is reconstructed by suture, so that the posterior wall of the inguinal canal is flattened (caveat: do not damage the structures running behind the transversalis fascia). **h** Mesh fixation from medial to lateral, here at Cooper’s ligament. **i** Absorbable suture fixation of the mesh with a loose knot to the iliac fascia (*needle in* *circle*) under safe avoidance of the nerves (*green arrows*). *M* medial hernia/transversalis fascia, *L* lateral hernia/inner inguinal ring

The surgical steps, which are divided into steps 1–8 here, are presented in the video in the same systematic approach and are as described below (Fig. 4):

### Step 1—Incision of the peritoneum (Fig. 4a; additional material online, video sequence 01:39 min).

Opening of the peritoneum from lateral to medial, starting at the level of the anterior superior iliac spine. This approach is made in a wide, wing-like arch in order to be able to position a sufficiently large mesh.

### Step 2—Exposure of the pubic symphysis (Fig. 4b; additional material online, video sequence 02:30 min).

Medial exposure of the rectus abdominis muscle (preserving the arcuate line and the fascia of the rectal muscle), the symphysis and the urinary bladder in the space of Retrzius (this approach differs from the TEP [total extraperitoneal hernia repair] technique, in which the entry is made through the posterior rectus sheath). The medial insertion of the inguinal ligament, the pectineal ligament and the lacunar ligament are visualized. The symphysis is sufficiently cleared to eventually allow the mesh to overlap approximately 2 cm to the contralateral side.

### Step 3—Visualization of the nerves under the iliac fascia (Fig. 4c; additional material online, video sequence 03:30 min).

Lateral visualization of the iliac fascia, with visualization of the nerves running underneath (lateral femoral cutaneous nerve and the genitofemoral nerve with its genital and femoral branches). This is where the Bogros’ space is located. In rare cases, the nerves do not run under the fascia; by starting the dissection from lateral, such atypical nerve courses can be reliably identified.

### Step 4—Preparation of the hernial orifices (Fig. 4d–g; additional material online, video sequence 04:08 min).

Preparation of the myopectineal orifice from lateral to medial. Mobilization of the lateral hernia sac (“inner sac”) from the inguinal canal, with separation of the same from the testicular vessels, which are surrounded by fat and loose connective tissue and the deferent duct (Fig. 4d). If the hernial sac is very long or embryologically attached to the testicle in the scrotum, the distal portion may be left in place. The inguinal canal must always be checked for a possible accompanying fat prolapse, the spermatic cord lipoma (Fig. 4e). In case of a large lateral hernia, the inguinal canal is sealed by spraying it with fibrin glue (Tisseel 4 ml, Baxter, with flexible applicator) to reduce the incidence of symptomatic seroma (Fig. 4f; additional material online, video sequence 08:41–09:17 min).

The medial hernia is then dissected medial to the epigastric vessels; due to the pressure of the pneumoperitoneum, the transversalis fascia bulges outwards (“outer sac”) when a medial hernia is present. The transversalis fascia is reconstructed with a continuous V‑Loc suture; the posterior wall of the inguinal canal is thereby flattened (Fig. 4g; additional material online, video sequence 09:27–10:53 min); it is essential to pay attention to the course of the testicular vessels and the deferent duct, which are located anterior to the transversalis fascia in the inguinal canal, so that they are not grasped “blindly” during suturing (Fig. 1/17’; caution: bleeding and chronic pain). Medial to the epigastric vessels and below the inguinal ligament, the femoral canal is cleared along the external iliac vein and examined for a femoral hernia (additional material online, video sequence 10:56–11:24 min).

At this point, attention is paid to the external iliac vessels which run in the triangle formed by the deferent duct and the testicular vessels, known as the “triangle of doom” (Fig. 1/*): careless dissection at this point can spell (bleeding) doom [16].

After completion of all these steps, the hernia is classified according to the EHS classification (L1–3, M1–3, and/or F1–3).

### Step 5—Dorsal parietalization (additional material online, video sequence 08:42 min).

The deferent duct is freed deep into the lesser pelvis. Now the parietalization is performed dorsocranially along the psoas and iliac muscles over a distance of at least 8 cm in the area of the space of Bogros. The nerves remain in the field of vision under the iliac fascia and are spared.

### Step 6—Insertion of the mesh (additional material online, video sequence 10:03 min).

Insertion of a mesh measuring at least 10 × 15 cm (large-pore, MRI-visible, Dynamesh, Aachen, Germany). The mesh positioning starts at the symphysis in the space of Retzius, the lower edge of the mesh lies laterally and dorsocranially in the Bogros’ space. The mesh should overlap the main hernia by about 5 cm. Depending on the findings, a larger mesh must be used (e.g., 12 × 17 cm). In case of bilateral hernia repair, the meshes overlap medially by 2–3 cm.

### Step 7—Mesh fixation (Fig. 4h, i; additional material online video, sequence 10:29 min).

Mesh fixation with loose absorbable sutures at 4 points. Helpful to keep the mesh without folds is to start (a) at the pectineal ligament (Fig. 4h), then (b) at the facia of the rectus muscle; (c) at the transversus abdominis muscle and finally (d) at the iliac fascia (Fig. 4i). Caution: this last point of fixation is formally located in the “Triangle of Pain”, the anatomical area lateral to the testicular vessels and inferior to the iliopubic tract (Fig. 1/**; [17]). The HerniaSurge Guideline strongly advises against stapler fixation at this site because of the risk of nerve injury. However, under robotic working conditions, fixation with a loose knot can be placed precisely to the iliac fascia with safe sparing of the nerves. We advocate this fixation because the recurrence of endoscopic repairs is usually found at this location. In femoral hernias, two nonabsorbable sutures are placed on Cooper’s ligament because of the limited range of dorsal mesh overlap.

### Step 8—Closure of the peritoneum (additional material online, video sequence 11:51 min).

Suture closure of the peritoneum from lateral to medial. When using monodirectional self-locking suture materials (e.g., V‑Loc/Medtronic Germany or Stratafix/Ethicon–Johnson&Johnson), it is essential to hide the suture stump at the end of the suture, otherwise intestinal adhesions will form on the suture stump, which can lead to ileus requiring revision as short as 2 days postoperatively [18]. The needles and suture remnants are removed from the abdomen and all surgical materials are counted. The fascial gaps in the area of the three 8 mm ports do not need to be closed. The skin is sutured intracutaneously with absorbable suture material and sealed with cyanoacrylate adhesive (alternatively hydrocolloid dressing for optimal tension relief).

## Postoperative care

Peri- and postoperative analgesia is very important to avoid the chronification of perioperative pain. Outpatients go from the operating room to the recovery room for 1–2 h and are then discharged via the day clinic, where they spend another 2–3 h. Before discharge, they stand with the help of the nurses, go to the toilet independently, and are given a light meal. Physical activities and sports are recommended according to the symptoms and as early as possible; a specific restriction of activities is not necessary [19]. The suture material does not need to be removed. Scar care with UV blocker for 6 months and a massage roller for the scars is usually appreciated by the patients.

## Casuistics and study design

This video article summarizes the experience of 302 consecutive r‑TAPPs that were performed over an 18-month period. It is a prospective cohort study with no control group. Data collection began with the first procedure of the implementation phase of the Visceral Surgery Robotics Program at Kantonsspital Olten (KSO) and, thus, includes the period of the learning curve in the use of the surgical robot. The study was approved by the ethics committee of Northwestern Switzerland (Ref. No. 2019-02046). Decisions on interventions at the level of the hernial orifices (suturing of the transversalis fascia or fibrin glue sealing of the inguinal canal) and mesh size were made intraoperatively depending on the findings in the sense of the tailored approach as part of the usual care order. All patients were followed up clinically and, if necessary, sonographically 6 weeks postoperatively. All data were recorded in a pseudonymized way in a hospital database, which is password-protected and accessible to the investigators only. Following Swiss legislation, unilateral operations were performed preferentially as outpatient surgery (ambulant-vor-stationär, AVOS). Bilateral inguinal hernia repairs, recurrent operations and operations in patients with an increased risk profile (e.g., oral anticoagulation, coagulation disorders or after previous prostatectomy) were treated as inpatient procedures. The procedural time over the three evaluation periods was analyzed with the analysis of variance (ANOVA) test; the reduction of the seroma rate was analyzed with the chi-square test for trend (GraphpadPrism, USA).

## Results

A total of 302 hernias were operated on in 225 patients (87.6% men; mean age 58.7 years). The demographic characteristics, concomitant diseases, and risk factors are listed in Table 1 in three time periods.

**Table 1:** Demographic data

	Total	First period	Second period	Third period
		05/2018–10/2018	11/2018–04/2019	05/2019–10/2019
	*n* = 225	*n* = 60	*n* = 87	*n* = 78
*Sex*				
Female	28 (12.4%)	4 (6.7%)	9 (10.3%)	15 (19.2%)
Male	197 (87.6%)	56 (93.3%)	78 (89.7%)	63 (80.8%)
*Age in years, mean (range)*	58.7 (19–95)	58.0 (19–85)	59.4 (24–85)	58.6 (23–95)
*BMI (kg/m*^*2*^*), mean (range)*	25.5 (16.3–42.6)	25.6 (17.9–34.6)	25.4 (16.3–34.3)	25.5 (17.0–42.6)
*Ethnicity*				
Central European	169 (75.1%)	44 (73.3%)	67 (77.0%)	58 (74.4%)
Mediterranean	56 (24.9%)	16 (26.7%)	20 (23.0%)	20 (25.6%)
*ASA Classification*				
1	51 (22.7%)	12 (20.0%)	16 (18.4%)	23 (29.5%)
2	144 (64.0%)	35 (58.3%)	63 (72.4%)	46 (59.0%)
3	20 (8.9%)	5 (8.3%)	7 (8.0%)	8 (10.3%)
Unknown	10 (4.4%)	8 (13.3%)	1 (1.1%)	1 (1.3%)
*Comorbidities*				
Arterial hypertension	87 (38.7%)	23 (38.3%)	37 (42.5%)	27 (34.6%)
Diabetes mellitus	19 (5.8%)	7 (11.7%)	7 (8.0%)	5 (6.4%)
COPD	7 (3.1%)	2 (3.3%)	3 (3.4%)	2 (2.6%)
Coronary heart disease	16 (7.1%)	5 (8.3%)	4 (4.6%)	7 (9.0%)
Nicotine abuse	67 (29.8%)	22 (36.7%)	26 (29.9%)	19 (24.4%)
Oral anticoagulant	8 (3.6%)	2 (3.3%)	3 (3.4%)	3 (3.8%)
Aspirin/Clopidogrel	30 (13.3%)	11 (18.3%)	12 (13.8%)	7 (9.0%)

*BMI* Body mass index, *COPD* chronic obstructive pulmonary disease, *ASA* American Society of Anesthesiologists

The majority of hernias operated on were primary hernias, with the proportion of recurrent hernias increasing to 21.8% in the third study period (Table 2). Every fourth patient had another finding on the operated side in addition to the previously clinically known inguinal hernia (femoral, obturator or Spieghel hernia). Among the inguinal hernias, large lateral hernias (L2) were found most frequently, followed by L1 and M2 hernias. The majority of hernias were treated with 10 × 15 cm mesh (90.4%). Larger meshes (12 × 17 cm) were used with increasing frequency over the 18 months (2.5% in the 1st period, 11.2% in the 3rd period). At the beginning, the meshes were rarely fixed; with increasing experience and full use of the fine motor possibilities of robotics, mesh fixation with absorbable suture with air knots at 4 points was adopted in the standard operating procedure (intraoperative checklist; Table 2). The operating time from incision to skin suture (including adhesiolysis in individual cases) was 71 min on average for unilateral hernias (range 40–186 min), 103 min for bilateral hernias (range 58–193 min) and 95 min for unilateral recurrent hernias (range 54–186 min). Overall, there was no difference in the procedural time across the three evaluation periods (*p* = 0.513). The time from the onset of pneumoperitoneum (incision) to the start of console work averaged 7 min (range 4–12 min). Unilateral suturing of the transversalis fascia took an average of 6:20 min (range 02:49–10:15 min). Unilateral fibrin glue sealing of the inguinal canal took an average of 03:47 min (range 2:17–04:53 min). Mesh fixation with 4 absorbable sutures took an average of 05:17 min (range 2:05–09:35 min).

**Table 2:** Hernia diagnosis, surgical data and hospital stay

	Total	First period	Second period	Third period
		05/2018–10/2018	11/2018–04/2019	05/2019–10/2019
	Patients *n* = 225	*n* = 60	*n* = 87	*n* = 78
*Hernia type*	302 (100%)	80 (100%)	115 (100%)	107 (100%)
Patients unilateral	148 (65.8%)	40 (66.7%)	59 (67.8%)	49 (62.8%)
Patients bilateral	77 (34.2%)	20 (33.3%)	28 (32.2%)	29 (37.2%)
*Primary groin hernia*	269 (89.1%)	75 (93.8%)	104 (90.4%)	90 (84.2%)
*Recurrent groin hernia*	33 (10.9%)	5 (6.2%)	11 (9.5%)	17 (15.8%)
*OR duration in min*^*a*^*, mean (range)*				
Unilateral repair	70.8 (40–186)	66.6 (40–94)	71.6 (41–131)	73.3 (45–186)
Bilateral repair	103.9 (58–193)	97.8 (68–159)	112.3 (75–193)	99.6 (58–140)
Recurrent groin hernia	95.5 (54–186)	90 (54–139)	98.9 (54–169)	94.9 (60–186)
After prostatectomy	86	86	–	–
*Teaching procedure (n)*	110 (48.9%)	12 (20.0%)	51 (58.6%)	47 (60.3%)
*Number of hernias per side*				
0 (no groin hernia)	1 (0.3%)	0	1 (0.9%)	0
1 (simple)	240 (79.5%)	64 (80.0%)	94 (81.7%)	82 (76.6%)
2 (combined)	47 (15.6%)	12 (15.0%)	17 (14.8%)	18 (16.8%)
3 (combined)	14 (4.6%)	4 (5.0%)	3 (2.6%)	7 (6.5%)
Other: obturator/Spieghelian	8 (2.6%)	1 (1.3%)	3 (2.6%)	4 (3.7%)
*EHS Classification*				
M1 + M2	85	26	27	32
M3	11	1	3	7
L1 + L2	212	57	80	75
L3	22	2	12	8
F1 + F2	43	12	13	18
F3	3	1	2	0
*Suture of transversalis fascia*^*b*^	55/96 (57.2%)	13/27 (48.14%)	18/30 (60.0%)	24/39 (61.5%)
*Fibrin glue sealing*^*c*^	15/324 (6.4%)	0	0	15/83 (18.0%)
*Mesh size*				
10 × 15 cm	278 (92.5%)	78 (97.5%)	105 (91.3%)	95 (88.8%)
12 × 17 cm	24 (7.9%)	2 (2.5%)	10 (8.7%)	12 (11.2%)
*Mesh fixation*^*d*^				
None	87 (28.8%)	70 (87.5%)	15 (13.0%)	2 (1.9%)
Absorbable sutures	215 (92.0%)	11 (13.5%)	100 (86.9%)	105 (98.1%)
*Duration of hospitalization*				
Day-care	80 (35.6%)	14 (23.3%)	40 (46.0%)	26 (33.3%)
1 night	99 (44.0%)	36 (60.0%)	29 (33.3%)	34 (43.6%)
2 nights	37 (16.4%)	7 (11.7%)	15 (17.2%)	15 (19.2%)
≥3 nights	9 (4.0%)	3 (5.0%)	3 (3.4%)	3 (3.8%)

*EHS* European Hernia Society, *OR* operating room

^a^Time measurement from the beginning of the installation of the pneumoperitoneum (including targeting of the DaVinci Xi), docking and surgery (including suturing of the transversalis fascia or fibrin glue sealing of the inguinal canal as well as mesh fixation) to the end of the skin suture. The time from the beginning of the pneumoperitoneum (incision) to the start of work on the console takes an average of 7 min (range 4–12). There is no significant difference in the overall procedure time between the analyzed periods (ANOVA, *p* = 0.513)

^b^Unilateral transversalis fascia suturing takes an average of 06:20 min (range 2:49–10:15)

^c^Unilateral fibrin glue sealing of the inguinal canal takes 03:47 min on average (range 02:17–04:53)

^d^Mesh fixation with 4 absorbable sutures takes 05:17 min on average (range 02:05–09:35)

In all, 48% of procedures were teaching procedures, in which residents performed parts of the procedure independently in the sense of “entrustable professional activities” [20]. The duration of the teaching procedures is included unabridged in the above-mentioned operating times (Table 2). The use of instruments was very constant for all procedures; in 98% of the cases only the three planned instruments monopolar scissors (Hot Shears MCS), grasping forceps (Prograsp Forceps), and needle holder (Mega SutureCut Needle Driver) were used.

A total of 14 patients (6.2%) refused to have a follow-up or did not show up. The average time to first follow-up was 41.4 days (range 1–168 days). The majority of patients (84.4%) required only one follow-up (Table 3). Postoperative complications are summarized in Table 3. Urinary retention requiring catheterization was observed in 8 cases. Seromas were described in 6.6% of cases and the majority were treated conservatively; the incidence of seroma was 11.2% in the first period and decreased to 3.0% in the third study period (Table 3). Seven cases underwent seroma aspiration in the outpatient clinic. One patient required an open seroma capsule resection 6 months after the index operation due to persistent symptoms and was subsequently symptom-free; in this patient, fibrin glue sealing of the inguinal canal had not been performed. Postoperative hematoma was observed in 10 cases (3.3%); 4 of these 10 patients were taking aspirin or clopidogrel (40% of hematoma cases), while among patients without hematoma only 7.8% were taking aspirin or clopidogrel; 2 patients underwent hematoma revision with drainage insertion under general anesthesia (0.6%). There were no wound complications and no postoperative ileus. None of the patients experienced pain due to nerve lesion (no neuropathic pain). No recurrence has occurred to date.

**Table 3:** Outcomes and complications

	Total	First period	Second period	Third period
		05/2018–10/2018	11/2018–04/2019	05/2019–10/2019
Patients (hernias)	*n* = 225 (302)	*n* = 60 (80)	*n* = 87 (115)	*n* = 78 (107)
*Follow-up 6 weeks*	211 (93.7%)	55 (91.6%)	80 (91.9%)	76 (97.4%)
Unplanned follow-up	9 (4.0%)	4 (6.7%)	3 (3.4%)	2 (2.6%)
*Complications, n (%)*				
*Urinary retention *(CD 2)	8 (3.6%)	–	4 (4.6%)	1
*Symptomatic seroma*^*a*^	20 (6.6%)	9 (11.2%)	7 (6.0%)	4 (3.6%)
Conservative (CD 1)	12 (60.0%)	6 (66.7%)	5 (71.4%)	1 (25.0%)
Aspiration (CD 3a)	7 (35.0%)	2 (22.2%)	2 (28.6%)	3 (75.0%)
Operation (CD 3b)	1 (0.3%)	1 (1.2%)	–	–
*Hematoma*	10 (3.3%)	5 (6.2%)	3 (2.6%)	2 (1.8%)
Conservative (CD 1)	8 (80.0%)	5	2	1
Operation (CD 3b)	2 (20.0%)	–	1	1
*Pulmonary embolism* (CD 4)	1 (0.3%)	–	–	1
*Deep vein thrombosis* (CD 2)	1 (0.3%)	–	1	–
*Epididymitis*	7 (2.3%)	4 (5.0%)	3 (2.6%)	–
Antibiotics (CD 2)	7	4	3	–
*Surgical site infection*	**–**	**–**	**–**	**–**
*Postoperative ileus*	**–**	**–**	**–**	**–**
*Recurrence*	**–**	**–**	**–**	**–**

*CD* Clavien–Dindo classification of surgical complications

^a^There is a significant trend to fewer seromas from the first to the last evaluation period (Chi-square test for trend, *p* = 0.043)

## Discussion

The advantages of the robotic system relevant to endoscopic r‑TAPP are as follows: to work in a larger intra-abdominal space even with low pneumoperitoneum pressure, standardized working distance to the target organ, immersion view and stable camera guidance, guidance of the precision instruments with 3:1 motion-transmission (which means that the choreography of the movement is gross motorized, but the execution is fine motorized) and finally the special value in advanced training of residents, with application of the dual operating console. The lack of haptics is far compensated by the positive balance of all the above listed advantages and is not an issue in everyday life. The expertise gained with the operation of the DaVinci Xi in the treatment of inguinal hernias has proven itself useful also in the performance of major visceral surgical procedures. In our clinic, in addition to deep anterior rectal resection with total mesorectal excision (TME), this also includes right oncological hemicolectomy with complete mesocolic excision (CME), gastrectomy with D2 lymphadenectomy or full wall gastric resections for gastrointestinal stromal tumor (GIST) and bariatric surgery, to name a few.

Comparisons of robotic and laparoscopic surgical techniques have been mostly equivalent in terms of surgical outcome, with a small advantage on reduced postoperative pain described after robotic surgery [21, 22]. Compared to open inguinal hernia repair, robot-assisted surgery showed a significantly lower number of postoperative complications and reoperations [23]. Postoperative seroma is a problem that has not yet been solved, and various strategies have been evaluated for its prophylaxis [24]. Morphological reconstruction of the posterior wall of the inguinal canal has historically mainly been bypassed in conventional endoscopic procedures for technical reasons, but this is no longer a particular challenge with robotics. Suture repair of the transversalis fascia has been performed more and more frequently in our collective and is currently standard, with the exception of very small findings. Pini et al. sutured the transversalis fascia with V‑Loc suture on 61 r-TAPP sites and observed neither seroma nor recurrence after 30 days, and no prolonged or chronic pain over a 10-month follow-up [25]. There is one small randomized trial of fibrin sealant in 40 patients, showing that seroma prevention is significant in the intervention group (*p* < 0.001; [26]). In the current series, 17 of 20 seromas were observed in lateral hernias, of which only 1 patient had been sealed with fibrin glue. Further randomized studies are needed to prove or reject the positive correlation of fibrin glue sealing on the reduction of seroma incidence.

Learning curves attempt to record the number of operations before an indicator (e.g., operating time) reaches a stable plateau. In the context of robotics in a teaching hospital, two learning curves always have to be mastered simultaneously: the procedure-specific learning curve (the “TAPP operation” itself) and mastery of the robot. The learning curve of r‑TAPP is steep and the described reductions in surgery time are proof of the rapid and intuitive adoption of the advantages of robotics [27, 28]. In a study of r‑TAPP from Italy, it was calculated that after 43 r-TAPPs operated by experienced surgeons, the learning curve reached the plateau (from 70 to 61 min; [29]). The r‑TAPP learning curve of the European pioneer of robotic hernia repair Filip Muysoms from Belgium, shows similar results: in one year the operating time (incision-to-skin-closure) was reduced from 80 to 60 min; in Muysoms et al. the average console time for unilateral hernias is 43 min (total operating time 94 min), for bilateral hernias 65 min (total operating time 119 min; [27]).

**Fig. 5:**
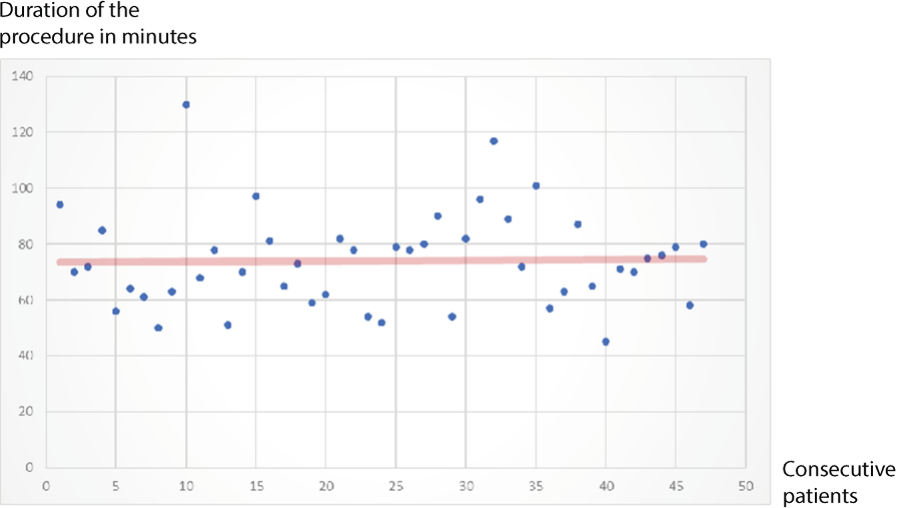
Learning curve of robotic inguinal hernia repair (r-TAPP) in the context of residents’ training with use of the double console. All consecutive cases of the study period are shown

From our data, the stable operating room time for resident’s training procedures with the double console is now apparent (Fig. 5). There may be three reasons for this extraordinary learning curve: (a) from the beginning of the robotics program, we decided to minimize the procedure-specific learning curve on the patient, (b) to complete the robotic learning curve on the simulator by requiring each surgeon to practice on the simulator for at least 20 h before starting on the console (at KSO, we have our own simulator on which the team is continuously trained), and (c) through division of labor in the sense of “entrustable professional activities”, residents may perform some steps during the operations, for example, the incision of the peritoneum, under supervision and alternating console handovers, then work on the hernial orifices several times in consecutive procedures, and again, mesh position and fixation suture also in several consecutive patients. One possible reason why the operating time of experienced surgeons is not further reduced is that surgeons spend more time in dialogue with the tissue, as more details are perceived and ergonomic challenges do not influence the speed of the procedure. A second reason is that more and more complex cases are operated on with the DaVinci Xi (Table 2). The time between two operations varies from 18–35 min depending on the comorbidities of the patient.

A limitation of the current study is the lack to date of long-term results on the incidence of recurrence; a more accurate interpretation of prolonged (nonneuropathic) postoperative pain, especially among non-Northern-European patients, is also pending. Another limitation is that although we observed fewer seromas with increasing expertise and adaptation of surgical technique, whether repair of the transversalis fascia and fibrin glue sealing have a positive effect on seroma reduction needs to be clarified in a future prospective randomized trial.

In Switzerland, under the actual extended use program of the instruments, the r‑TAPP incurs additional costs of 950 SFr for the material and 420 SFr for the maintenance flat rate of the robot (considering 300 procedures/year) per patient. These costs may be comparable to Europe and the USA at an exchange rate of approx. 1:1 for the euro or US $. With, for example, 10,000 procedures per year and 8 million residents with statutory health insurance in Switzerland, the robotic treatment of all inguinal hernias would burden the general public with an additional amount of 14 cent/month or 1.71 SFr/year, or the equivalent of a half a cup of coffee/year/insured person. Future studies are needed to show whether the improvement in outcomes expected from robotics is reproducible; if so, these would not only compensate for the cost but, more importantly, positively affect the quality of life of the individual patient.

To argue that r‑TAPP is unnecessary because it has no advantages at a first glance and that it is too expensive shows either ignorance of the method or denial of the historical assertiveness of technological advances, especially in visceral surgery [30]. Surgery of inguinal hernias will almost certainly never reach a conclusive chapter. First, because the reparative intervention on hernia will hardly take place on the genome level in a laboratory but will always remain an anatomical–surgical intervention; second, progress continues inexorably and more precise instruments will continue to improve the handling of the tissue again and again; and finally, the heterogeneity of the hernia findings will defy a premature conclusion of this chapter for generations to come.

## Conclusions

- Advanced anatomical knowledge of the myopectineal orifice is essential for robotic inguinal hernia repair (r-TAPP).
- Suturing of the transversalis fascia, fibrin glue sealing of the inguinal canal, and suture fixation of the mesh are additional steps whose added value for the outcome must be demonstrated in future studies.
- Working with a double surgical console offers optimal conditions for residents training, minimizing the procedure-related learning curve on the patient, and observing operating times suitable with the narrow schedules of the operating room.
- The postoperative seroma formation and complication rate of r‑TAPP are low.
- The r‑TAPP is the natural evolution of the conventional TAPP, and its acceptance will grow proportionally to equipment availability and cost reduction.

## Supplementary Information

Video: Robotic inguinal hernia repair (r-TAPP) in 8 steps

## Declarations

The data collection of this study is based on a positive ethics vote of the Ethics Committee Northwestern Switzerland (No. 2019-02046).
