# Erratum to: Robotic hernia surgery I. English version: Robotic inguinal hernia repair (r-TAPP). Video report and results of a series of 302 hernia operations

**DOI:** 10.1007/s00104-021-01562-y

**Published:** 2021-12-20

**Authors:** Michaela Ramser, Johannes Baur, Nicola Keller, Jan F. Kukleta, Jörg Dörfer, Armin Wiegering, Lukas Eisner, Ulrich A. Dietz

**Affiliations:** 1 Department of Visceral, Vascular and Thoracic Surgery, Cantonal Hospital Olten (soH), Baslerstrasse 150, 4600 Olten, Switzerland; 2 Department of General, Visceral and Vascular Surgery, Cantonal Hospital Baden, Im Engel 1, 5404 Baden, Switzerland; 3 Hernienzentrum Zurich, Grossmuensterplatz 9, 8001 Zurich, Switzerland; 4 Department of General, Visceral, Transplant, Vascular and Pediatric Surgery, University Hospital Wuerzburg, Oberduerrbacher Straße 6, 97080 Wuerzburg, Germany

**Erratum to:**

**Chirurg 2021**

10.1007/s00104-021-01446-1

The article “Robotic hernia surgery.r-TAPP” is part I and a translation of the originally in *Der Chirurg* 8/21 published German language article. The title and subtitle have been amended accordingly. The original article has been corrected.

